# Radiographic findings in tuberculous diabetic patients

**DOI:** 10.4103/0970-2113.76309

**Published:** 2011

**Authors:** Akashdeep Singh

**Affiliations:** *Department of Pulmonary Medicine, Christian Medical College and Hospital, Ludhiana, Punjab, India. E-mail: drsinghakashdeep@gmail.com*

The association between diabetes mellitus and tuberculosis has been recognized for centuries. In recent decades, tuberculosis incidence has declined in high-income countries, but incidence remains high in countries that have high rates of infection with HIV, high prevalence of malnutrition and crowded living conditions, or poor tuberculosis control infrastructure. At the same time, diabetes mellitus prevalence is rising globally, fuelled by obesity. There is growing evidence that diabetes mellitus is an important risk factor for tuberculosis and might affect disease presentation and treatment response. Furthermore, tuberculosis might induce glucose intolerance and worsen glycemic control in people with diabetes.

The radiographic presentation of tuberculosis depends on many factors, including duration of illness and host immune status. In 1927, Sosman and Steidl[1] reported that a large proportion of diabetic patients with tuberculosis had lower lung involvement, whereas nondiabetic patients usually had upper lobe infiltrates. Subsequent studies in the 1970s and 1980s corroborated this finding,[2,3] and it was widely believed that pulmonary tuberculosis in diabetic patients presented with an atypical radiographic pattern and distribution, particularly lower lung involvement. Clinically, this is important because lower lobe tuberculosis might be misdiagnosed as community-acquired pneumonia or cancer. A high degree of suspicion is required, especially in a diabetic patient who develops lower lobe opacities.

Furthermore, patients with pulmonary tuberculosis that do not have upper lobe involvement are less likely to have positive sputum smears and cultures.[4]

Judicious and early planning of fibreoptic bronchoscopy combined with transbronchial lung biopsy may clinch the diagnosis in a significant number of such cases.

In some series, multilobar disease or the presence of multiple cavities was more common in diabetic patients, but lower lung disease was rarely more common in diabetic patients than in controls, except, perhaps, in patients aged over 40 years.[4–7]

Prognosis of PTB infection is good if diagnosed and treated early; together with control of underlying condition. The clinicians should be aware of atypical radiological manifestations of the tuberculosis when coexisting with diabetes mellitus.
